# Occupation-Related Injuries Among Healthcare Workers: Incidence, Risk Groups, and the Effect of Training

**DOI:** 10.7759/cureus.14318

**Published:** 2021-04-06

**Authors:** Buket Erturk Sengel, Elif Tukenmez Tigen, Huseyin Bilgin, Arzu Dogru, Volkan Korten

**Affiliations:** 1 Infectious Diseases and Clinical Microbiology, Marmara University School of Medicine, Istanbul, TUR; 2 Infectious Diseases and Clinical Microbiology, Tuzla Training Hospital, Istanbul, TUR

**Keywords:** blood and body fluid, healthcare workers, needlestick and sharp-object injury, occupational

## Abstract

Background and objective

Occupation-related injuries (ORIs) are undesirable and harmful situations among healthcare workers (HCWs) and may have serious consequences. In this study, we aimed to identify and analyze ORI incidences, risk groups, and the outcomes of a training program to prevent them.

Materials and methods

Between January 2011 and December 2019, HCWs who applied for infection prevention and control (IPC) due to ORIs (percutaneous needlestick and sharp-object injury or contact with blood or body fluids) were included in the study. Their characteristic features, vaccine histories, injury types, viral serologies, and administered prophylaxis were recorded. After 2014, a periodic ORI training program was started. We used joinpoint regression analysis to compare the ORI incidences before and after the education program.

Results

During the nine-year study period, 965 ORIs were registered. The mean age of HCWs was 39.3 ± 8.4 years, and 67.9% of them were female. The total injury incidence for all professions was 34.1 (95% CI: 33.1-37.5) per 1,000 HCWs. The injury incidences were significantly higher in nurses compared to other HCWs (p<0.01). Most of the injuries occurred in the ward setting (37%). HCWs were injured most commonly while administering treatment (36.7%). The trend analysis for the incidence of injuries showed no significant change throughout the study period. The trend in personal protective equipment (PPE) use showed a significant increase (annual percentage change: 1.7, p<0.01).

Conclusions

The major finding of this study with respect to its implication on the healthcare system is that nurses are an important risk group for ORIs. Although the ORI incidence did not change during the study period, a significantly increased use of appropriate PPE following a systematic training program implementation was observed.

## Introduction

Harmful occupational events such as percutaneous needle stick injury (NSI) or injury from sharp objects and contact of mucous membrane and nonintact skin with blood or body fluids (BBFs) are common concerns among healthcare workers (HCWs) worldwide. The rates of bloodborne infection due to NSIs have been found to vary in different studies, with a high rate reported from developing countries due to the high prevalence of infections and insufficient adherence to standard precautions [1-3]. Although many kinds of pathogens have been identified, the potentially life-threatening occupation-related bloodborne pathogens are the hepatitis B virus (HBV), hepatitis C virus (HCV), and human immunodeficiency virus (HIV) [4]. According to the World Health Organization (WHO), it is estimated that 66,000 HBV, 16,000 HCV, and 1,000 HIV cases may occur annually among HCWs due to NSIs [5]. The risk of transmission following NSI is 6-30% for HBV, 0-7% for HCV, and <0.3% for HIV [6]. In addition to the life-threatening outcomes caused by these pathogens, there has been a significant associated increase in fear and anxiety among HCWs, apart from the increase in the financial burden to the healthcare system due to the surveillance and postexposure prophylaxis (PEP) that are set up to combat these types of injuries [7]. Therefore, we believe that serious efforts should be directed toward preventing undesired occupation-related injuries (ORIs) among HCWs. There are many strategies to prevent infections due to ORIs, such as the reduction of invasive procedures, periodic training of HCWs, and the use of safer devices during exposure to BBFs [8]. Nevertheless, if exposure to potentially infectious material occurs, it is crucial to appropriately treat and follow up on the HCWs.

NSIs are responsible for 86% of all occupation-related infections [9]. The most common causes of NSIs are two-handed recapping, drawing of blood, picking up waste, inserting a catheter, and performing surgeries [10].

In each hospital, there should be an ORI registry and training program for HCWs. However, even when these measures are in place, ORIs may not be monitored and managed properly because of the time-consuming procedures, lack of concern and attention, and the fear felt by injured HCWs regarding the possibility of positive test results [7,11]. Therefore, the true incidence rate of percutaneous injury and contact with BBF is not known. The rates of underreporting range from 13-85% in various countries [10,12-14].

In Turkey, there is no national recording system that documents the frequency of ORIs. The aim of this study was to determine the annual incidence of ORIs at the Marmara University Pendik Training and Research Hospital in Istanbul, Turkey. Secondary objectives were to define the characteristics of the injuries regarding the following parameters: (1) professional groups of HCWs and work sites at the time of ORI, (2) injury type, (3) time to admission after exposure, (4) use of personal protective equipment (PPE), (5) postexposure management and serological results of HCWs, and (6) effect of a periodic training program on the incidence of ORIs.

## Materials and methods

This retrospective study was conducted between January 2011 and December 2019 among HCWs at the Marmara University Pendik Training and Research Hospital, which is a 650-bed tertiary care center in Istanbul, Turkey. The collected data did not include any patient- and employee-identifying information. A waiver of informed consent was issued by the Marmara University School of Medicine Institutional Ethical Review Board (reference number: 09.2020.352). All HCWs including physicians, nurses, and others (laboratory technicians and housekeeping personnel) who applied for infection prevention and control (IPC) due to percutaneous injury (by NSI and/or a sharp object) and/or contact of mucous membranes and nonintact skin with BBFs were enrolled in the study. Nursing students and interns were not included since they are not allowed to perform invasive procedures at the hospital. The IPC team fills out an injury form for each person when they apply and inform them about the follow-up dates based on the source serology. We obtained data related to the following parameters from these records: gender, age, and profession; work sites at the time of ORI; injury type; procedures being performed at the time of the injury; time to admission for IPC; serological status of HBV, HCV, and HIV infections at the time of the injury and after the follow-up; use of PEP against HBV or HIV; usage of PPE; history of previous injuries; and occurrence of postexposure infection. Appropriate PPE use was defined as the presence of necessary PPE on the involved injury site at the time of ORI (e.g., the presence of gloves during an NSI of the hand).

After 2014, a training program was developed to target and improve the knowledge and practices and modify the behavior of HCWs, particularly the inexperienced among them and newly recruited personnel. A 30-minute interactive session with HCWs was carried out to cover topics such as standard precautions, strategies to prevent ORIs (avoiding two-handed recapping, handling, and disposal of sharp objects), the importance of reporting of ORIs, and usage of PPE. In these sessions, the IPC team explained to the HCWs the procedures to be followed in case of an injury. This training was repeated yearly, and extra sessions were conducted for newly recruited HCWs. During the routine IPC rounds, the team reminded the HCWs about strategies to prevent ORIs. In case of the occurrence of an injury, one-on-one education was given to the injured HCW about avoiding further injuries in the future.

A medical consultation was provided to all HCWs who had experienced an ORI; such consultations were available 24 hours a day and seven days a week.

Definitions

The treatment, which was one of the procedures being performed during the injury, was defined as intravenous (IV) interventions, catheter manipulations, dressing changes, bedside invasive procedures, or minor surgical procedures performed in the clinic.

While all injuries by a needlestick or sharp objects were defined as percutaneous injury, contact of mucous membrane and nonintact skin with BBFs without percutaneous injury was defined as mucocutaneous BBF exposure. The term “NSI” was just meant to signify an injury by a needle. Each ORI occurrence was considered a separate instance of injury. HCWs who were injured on more than one occasion were included.

HCWs who had not completed three doses of HBV vaccination were considered to be unvaccinated. The serological tests from HCWs who were exposed to HBV, HCV, and HIV-positive sources were obtained at the time of the injury and repeated at one, three, and six months. Complete postexposure follow-up was defined as the attendance in all three post-ORI follow-ups.

Statistical analysis

The sample size was calculated by determining the underreported case ratio to be 40% and the estimated injury incidence as 8% with 90% power and a 95% confidence interval. The estimated sample size was 595 injuries. Descriptive variables were presented as frequencies, percentages, means, standard deviations, medians, and percentiles. Categorical variables were compared with chi-square and Fisher’s exact analyses. The incidence of the injury was calculated on the basis of the number of injuries per 1,000 HCWs with a 95% confidence interval for the given year. To identify changes in injury incidence trends, joinpoint regression was estimated for each year group by using the Joinpoint Regression Program, Version 4.5.0.1 (Statistical Research and Applications Branch, National Cancer Institute, Bethesda, MD). By using incidences as inputs, this method identifies the year(s) when a trend change is produced, calculates the annual percentage change (APC) in incidences between trend-change points, and estimates the average annual percentage change (AAPC) during the whole study period. The type-1 error for the significant difference was 5%. Data were analyzed using the SPSS Statistics 22.0 (IBM, Armonk, NY) program.

## Results

In this study, the mean number of HCWs in the hospital was 3,036 ± 253 during the study period. The distribution of physicians, nurses, and other HCWs was 1,066 ± 67.0, 649 ± 196, and 1,429 ± 128, respectively.

During the nine-year study period, 965 injuries were registered. The mean age of the injured was 30.5 ± 8.2 years, and 68.4% of them were female. When categorized by profession, 59%, 86%, and 51% of physicians, nurses, and others were female, respectively. Age categorized into decades of lives revealed that 59.7% of injuries occurred in subjects between 20 and 30 years of age. In total, 47.4% of injuries were detected among nurses. HCWs were injured during treatments (36.7%), waste disposal (33.4%), and recapping (20.4%). Blood was the most commonly exposed body fluid (96.6%). HCWs were admitted to IPC in the first 24, 48, and 72 hours in 67.6% (652), 83% (801), and 90.1% of the cases, respectively. PPE was present in 88.4% of the injuries. In total, gloves were present in 86.9%, the gown was present in 13.1%, and the face shield was in place in 10.2% of the injuries. After the implementation of the training program, the glove use increased from 84.2% to 89.0% (p<0.03), face shield equipment use increased from 7.8% to 11.9% (p=0.04), but gown use did not change significantly (from 14.8% to 11.7%; p=0.17). In 24.2% of the cases, the HCWs had a history of the previous injury. All characteristic features of the HCWs are summarized in Table 1. The details of HCWs by their gender and profession are presented in Table 2.

**Table 1:** Characteristics of injured HCWs SD: standard deviation; BBF: blood or body fluids; HCWs: healthcare workers; ORI: occupation-related injury; n: number of HCWs

General characteristics	Values
Female gender, n (%)	660 (68.4%)
Age in years, mean ± SD	30.5 ± 8.2
Profession	
Nurses, n (%)	457 (47.4%)
Housekeeping personnel, n (%)	310 (32.1%)
Physicians, n (%)	163 (16.9%)
Laboratory technicians, n (%)	35 (3.6%)
Worksites at the time of ORI	
Ward, n (%)	359 (37.2%)
Intensive care unit, n (%)	183 (19%)
Emergency department, n (%)	143 (14.8%)
Operation room, n (%)	153 (15.9%)
Laboratory, n (%)	73 (7.6%)
Other locations in the hospital, n (%)	54 (5.6%)
Injury type	
Needlestick, n (%)	757 (78.4%)
Sharp objects, n (%)	85 (8.8%)
Mucocutaneous BBF, n (%)	71 (7.4%)
Other, n (%)	52 (5.4%)
Procedure during injury	
Treatment, n (%)	354 (36.7%)
Waste disposal, n (%)	322 (33.4%)
Recapping, n (%)	197 (20.4%)
Surgery, n (%)	92 (9.5%)
Age categories (years)	
20-30, n (%)	576 (59.7%)
31-40, n (%)	274 (28.4%)
41-50, n (%)	89 (9.2%)
>50, n (%)	26 (2.7%)

**Table 2:** Details of HCWs based on gender and profession (n=965) HCWs: healthcare workers

Gender	Profession			Total
	Physician, n (%)	Nurse, n (%)	Others, n (%)	N (%)
Male	67 (22%)	64 (21%)	214 (57%)	345 (100%)
Female	96 (14.5%)	393 (59.5%)	171 (25.9%)	660 (100%)
Total	163 (16.9%)	457 (47.4%)	345 (35.7%)	965 (100%)

**Figure 1:**
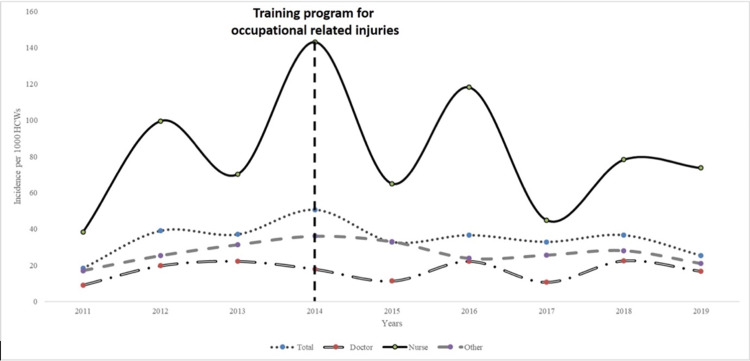
Incidences of ORIs by professions and years HCWs: healthcare workers; ORI: occupation-related injury

**Figure 2:**
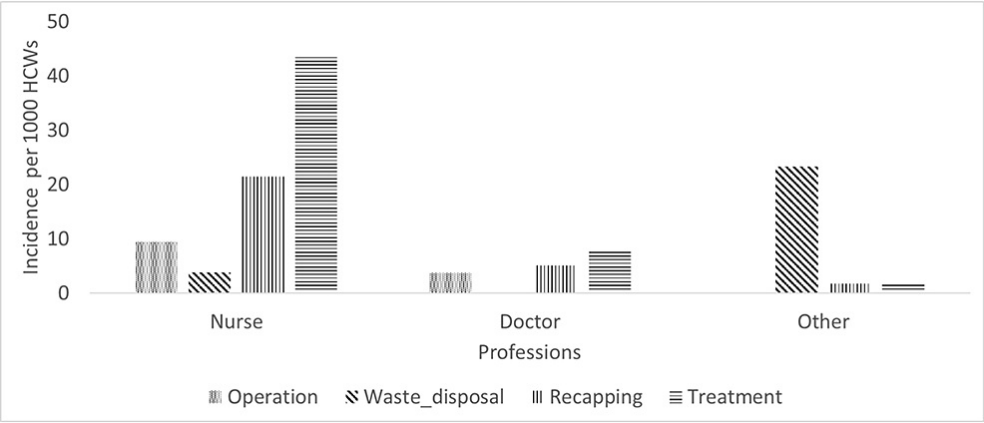
ORI incidence in different professions by procedures HCWs: healthcare workers; ORI: occupation-related injury

**Figure 3:**
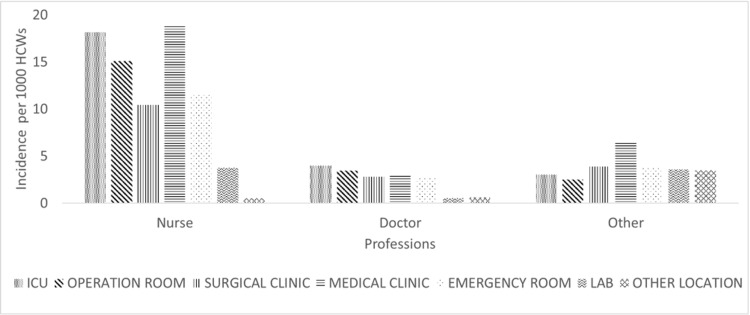
ORI incidence in different professions by worksites at the time of injury HCWs: healthcare workers; ORI: occupation-related injury; ICU: intensive care unit

The total injury incidence for all professions during the study period was 34.1 (95% CI: 33.1-37.5) per 1,000 HCWs. The highest injury incidence was recorded for nurses, specifically, 78.2 (95% CI: 71.3-85.6) per 1,000 nurses, followed by 26.8 (95% CI: 24.1-29.7) per 1,000 housekeeping and laboratory technicians and 16.9 (CI: 14.5-19.7) per 1000 physicians. The total injury incidences were significantly higher in nursing than in the remaining professions (p<0.01). We have shown the ORI incidence in different professions by years, procedures, and worksites of HCWs in Figures 1, 2, 3, respectively.

The most frequent exposure type among all HCWs was percutaneous injury (88.2%). The NSI was the most common type of injury among nurses (79.9%), followed by injury from other sharp objects (9.0%) and mucocutaneous BBF exposure (7.7%). The odds ratio of getting injured during the recapping procedure was 1.9 (95% CI: 1.4-2.5, p<0.01) for nurses compared to other professionals.

**Figure 4:**
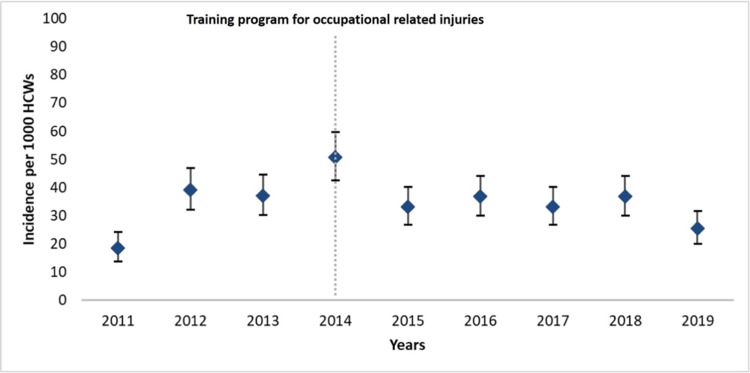
Yearly incidence of injuries per 1,000 HCWs The upper and lower bars represent the 95% confidence intervals. The trend of injury incidence did not differ significantly (p=0.46) HCWs: healthcare workers

After implementing the training program, both the incidences by profession and by year did not change significantly (Figure 1). Yearly total injury incidences are also shown in Figure 4. Injury rates by professions and procedures at the time of ORI before and after training were similar (Table 3). However, PPE usage showed a significant increase (APC: 1.7, p<0.01).

In total, 86% of the HCWs were either immune or vaccinated against HBV at the time of the injury. In 68 (7%) injuries, the source was hepatitis B surface antigen. In 12 (17.6%) of these 68 exposures, the HCWs were anti-HBs negative, and specific immunoglobulin and vaccines were applied to these HCWs. No seroconversion occurred in seven of these 12 HCWs. However, five HCWs did not complete their follow-up. Exposures to HCV- and HIV-positive sources occurred in 34 (3.5%) and 12 (1.1%) of the injuries, respectively. All 12 HCWs who were exposed to HIV-positive sources used antiretroviral prophylaxis. None of the patients with complete follow-up developed seroconversion for HIV and HCV during the postexposure follow-up. However, 240 (25%) of all HCWs did not complete the postexposure follow-up.

**Table 3:** Injury rates by professions and procedures at the time of ORI before and after training HCWs: healthcare workers; ORI: occupation-related injury

	Injury rates, n (%)		
Categories	Before training	After training	P-value
Nurses	190 (46.2%)	267 (48.2%)	0.55
Doctors	73 (17.8%)	90 (16.2%)	0.54
Other HCWs	148 (36.0%)	197 (35.7%)	0.07
Procedure during injury			
Recapping	94 (22.9%)	103 (18.6%)	0.10
Waste disposal	150 (36.5%)	172 (31.0%)	0.08
Treatment	134 (32.6%)	220 (39.7%)	0.02
Operation	33 (8.0%)	59 (10.6%)	0.18

## Discussion

ORIs pose the greatest risk of serious blood-borne infections for HCWs. Overall, the incidence of ORIs among HCWs ranges from 14 to 95 per 1,000 HCWs as per various studies from different countries [4]. The ORI incidence in our study was 34.1 per 1,000 HCWs, which is in line with the previous studies. In the literature, a significant proportion of reported NSIs has occurred during IV-related procedures or injections [7,15,16]. Similarly, NSIs were seen most commonly during treatment, including IV interventions and catheter manipulations in our study. In addition, many studies have shown that the prevalence of NSIs was more common in nurses than in other HCWs [15,17-19]. Garus-Pakowska et al. and Chaiwarith et al. found that blood collection has been the most frequent reason for injuries in nurses [15,20]. The more frequent injury rate in nurses can be explained by the fact that nurses had the most frequent contact with patients and these procedures were mostly done by nurses. In alignment with the existing literature, we recorded the highest injury incidence among nurses in our study (78.2% per 1,000 nurses).

The second most common procedure during an injury was waste disposal. The second most common group of injured HCWs comprised housekeeping personnel. The majority of injuries in housekeeping personnel occurred during waste disposal due to improper waste management (90.6%).

Various studies have shown that two-handed recapping, which is a common practice among HCWs, is the most common cause of NSIs [21,22]. Dilie et al. showed that HCWs who recap needles were 21.3 times more likely to experience NSI [18]. Therefore, avoiding recapping is critical to reducing the prevalence of NSIs. In our study, this accounted for 20.4% of all injuries. We observed that the highest injury incidence occurred in nurses, and compared with other professionals, they had 1.9 times the incidence of injury by recapping. One reason for the persistence of recapping behavior may be a lack of knowledge. Although recapping is a bad practice and not a nursing or medical standard, it is routinely practiced by HCWs. They may feel that recapping is a safe way to avoid NSIs or do not commonly use sharps containers in their practice.

Many studies have shown that occupational injuries decrease significantly with the implementation of strict preventive measures [17,23]. In particular, periodic training regarding standard precautions (information about PPE, avoiding two-handed recapping, vaccination, the importance of recording and using PEP, waste disposal) can help to reduce injuries [15]. However, the trend of overall ORI incidence showed no significant change after the implementation of the periodic training program in our study. One reason could be the high turnover of inexperienced HCWs in the hospital despite the periodical training program. Some HCWs leave their jobs, and new ones are hired every year. Even if they are educated, they may not gain professional experience in a short period of time. In addition, training alone may not be enough to prevent ORIs. We observed that the rate of injury during treatment actually increased after training. This may also be explained by the reasons mentioned above. Nearly 60% of HCWs are in the 20-30 year age category. Therefore, they may lack experience in performing invasive procedures such as treatment administration. Inexperienced HCWs may be stressed during treatment administration. Another reason may be that our center is a university hospital and has a high workload. In a study, it was found that higher workloads were associated with 50% to two-fold increases in the likelihood of NSIs [24].

A prospective study from the United Kingdom showed that educational programs alone did not change the NSI incidence [25]. Hence, multimodal approaches are needed. They showed that the combination of training with the provision of safety needle devices provided a significant reduction in NSIs. A meta-analysis involving 17 studies showed that a combination of training and the use of needle safety devices is more effective in reducing NSIs than either intervention alone [26]. However, due to a lack of resources in our center, we could not use needle safety devices, and therefore, we could not assess its effect on NSIs. Although the incidence of injury did not change, the use of PPE (especially glove and face shield usage) increased significantly in our study. These results indicate that systematic training programs can cause behavioral changes and subsequently increase PPE use in HCWs. Unfortunately, a training program alone was insufficient to improve the injury incidence. Therefore, it is important to combine different strategies to prevent ORIs in healthcare settings. Centers for Disease Control and Prevention (CDC) suggest using administrative controls, engineering controls (needle safety devices), PPE use, and workplace practice control methods [6]. Adams et al. have also shown that combining training and needle safety device use is a more effective strategy than training alone to prevent ORIs [25].

Unfortunately, we determined that 14% of HCWs were non-immune or unvaccinated against HBV at the time of injury. This rate is relatively high, but this may be because HCWs who have not completed three doses of vaccine are considered unvaccinated, HCWs who resist the vaccine, and some of them may be non-responders. Five out of 12 HBV-seronegative HCWs who were exposed to HBV-positive sources did not complete their follow-ups. However, all HCWs who contracted HIV- and HCV-positive sources completed their follow-up. The reason for this may be that HCWs are more afraid of HIV and HCV transmissions.

In the current study, the majority of ORIs were reported from general wards (47.2%), followed by ICUs (19%), emergency departments (EDs) (14.8%), and operating rooms (15.9%). These results are inconsistent with the current literature. Since workers in EDs, ICUs, and operating rooms need to act quickly in many cases, it may be assumed that the risks of injuries are higher in these departments. Besides, the HCWs in ED may engage in more work in a short period of time. In parallel, Amini et al. showed that NSIs occur most commonly in the ED [23]. In other studies, operating rooms have been reported as a common working site related to ORIs [27,28]. Our results may be due to underreporting by HCWs working in these areas owing to heavy clinical workload or lack of knowledge about reporting the injuries. Another reason for underreporting could be that screening for HBV, HCV, and HIV before any invasive procedure is a common practice in the country. In case of an injury, HCWs can check the source results without consulting IPC. If the viral serologies of the source are negative, the HCWs may not report their ORIs. Similar reasons can explain the late admissions after exposure (48 and 72 hours) to the IPC.

Underreporting is also a significant problem among HCWs [29]. Amini et al. and Garus-Pakowska et al. found underreporting rates among HCWs to be 50.2% and 45.2%, respectively [23,30]. The reasons for not reporting may be due to forgetting the injury, underestimation of the exposure risk, lack of awareness about reporting procedures, fear of positive serological test results, and time constraints [17,23].

There are some limitations to this study. Firstly, the data were collected retrospectively from injury records. Therefore, only reported injuries were considered. Secondly, we could not evaluate the association of ORIs with the length of time in the job among HCWs because of unavailable data. Some studies have demonstrated that short working experience is associated with a higher risk of ORIs [18,21]. Thirdly, we could not determine the number of HCWs who left or were hired year by year. Finally, we could not assess the financial burden caused by ORIs in the healthcare system.

## Conclusions

The present study has reinforced the fact that ORIs are still a significant concern among HCWs. Systematic training programs are necessary and should be conducted more frequently, but they alone are not adequate to reduce the incidence of ORIs. We suggest ensuring that the turnover rate among HCWs is reduced (e.g., by offering them permanent employment), thereby providing them the opportunity to gain more experience. Furthermore, the training programs should be accompanied by encouragement of the use of needle safety devices, and HCWs should be educated about avoiding underreporting of ORIs.
